# Lessons From an Outbreak of Legionnaires’ Disease on a Hematology-Oncology Unit

**DOI:** 10.1017/ice.2016.281

**Published:** 2016-12-06

**Authors:** Louise K. Francois Watkins, Karrie-Ann E. Toews, Aaron M. Harris, Sherri Davidson, Stephanie Ayers-Millsap, Claressa E. Lucas, Brian C. Hubbard, Natalia A. Kozak-Muiznieks, Edward Khan, Preeta K. Kutty

**Affiliations:** 1 Epidemic Intelligence Service Program, Atlanta, Georgia; 2 Centers for Disease Control and Prevention, Atlanta, Georgia; 3 Alabama Department of Public Health, Montgomery, Alabama; 4 Jefferson County Department of Health, Birmingham, Alabama

## Abstract

**OBJECTIVES:**

To define the scope of an outbreak of Legionnaires’ disease (LD), to identify the source, and to stop transmission.

**DESIGN AND SETTING:**

Epidemiologic investigation of an LD outbreak among patients and a visitor exposed to a newly constructed hematology-oncology unit.

**METHODS:**

An LD case was defined as radiographically confirmed pneumonia in a person with positive urinary antigen testing and/or respiratory culture for *Legionella* and exposure to the hematology-oncology unit after February 20, 2014. Cases were classified as definitely or probably healthcare-associated based on whether they were exposed to the unit for all or part of the incubation period (2–10 days). We conducted an environmental assessment and collected water samples for culture. Clinical and environmental isolates were compared by monoclonal antibody (MAb) and sequence-based typing.

**RESULTS:**

Over a 12-week period, 10 cases were identified, including 6 definite and 4 probable cases. Environmental sampling revealed *Legionella pneumophila* serogroup 1 (Lp1) in the potable water at 9 of 10 unit sites (90%), including all patient rooms tested. The 3 clinical isolates were identical to environmental isolates from the unit (MAb2-positive, sequence type ST36). No cases occurred with exposure after the implementation of water restrictions followed by point-of-use filters.

**CONCLUSIONS:**

Contamination of the unit’s potable water system with Lp1 strain ST36 was the likely source of this outbreak. Healthcare providers should routinely test patients who develop pneumonia at least 2 days after hospital admission for LD. A single case of LD that is definitely healthcare associated should prompt a full investigation.

Legionnaires’ disease (LD) is a severe form of pneumonia caused by gram-negative *Legionella* bacteria.^1^ *Legionella* species are ubiquitous in natural aquatic environments,^2^ but illness typically occurs when contaminated droplets of aerosolized water from a human-made source are inhaled or aspirated. Most reported cases are community acquired, but at least 7% are healthcare associated^3^; mortality in healthcare-associated LD is higher than in community-acquired cases and ranges from 13% to 46%.^4–6^ Patients with immune compromise are at increased risk of developing LD if exposed to *Legionella*^7^ and may experience increased severity and fatal outcomes,^8,9^ making prompt detection of outbreaks in healthcare facilities particularly important.

In May 2014, the Alabama Department of Public Health (ADPH) notified the Centers for Disease Control and Prevention (CDC) of 8 LD cases diagnosed since March 2014 among inpatients on a single hematology-oncology unit at an Alabama hospital. In this report, we characterize the outbreak, discuss contributing factors, and identify lessons learned.

## METHODS

### Outbreak Setting

The outbreak occurred at a medical center that serves as a hematology-oncology referral center for patients throughout the state. The hematology-oncology unit contains 27 single-occupancy patient rooms and occupies half a floor in the affected building, a 9-story building with independent water and ventilation systems. Although construction on most of the affected building was completed in 2009, construction of the hematology-oncology unit was not completed until December 2013; patients were first admitted on February 20, 2014.

### Case Definitions

A healthcare-associated LD case was defined as clinically or radiographically confirmed pneumonia and a positive urinary antigen test and/or respiratory culture for *Legionella* in a person with exposure to the hematology-oncology unit after February 20, 2014, and during the typical incubation period of 2–10 days prior to symptom onset. Definitely healthcare-associated LD cases occurred in patients admitted to the unit for the entire incubation period and in persons with a respiratory isolate identical to an environmental isolate from the unit determined by molecular sequence typing. Most likely, healthcare-associated cases occurred in persons with exposure to the unit for a portion of the incubation period with no clinical isolate.

### Case Finding and Chart Review

To identify potential LD cases, hospital laboratory records were reviewed for all respiratory cultures and urine antigen tests positive for *Legionella* since February 20, 2014. No *Legionella*-specific paired serology, direct fluorescence antibody testing, or PCR-based testing was performed during this period. In addition, ADPH reviewed all reported LD cases in the state since the unit opened for possible connection to the hospital. Charts of patients with a positive *Legionella* lab test and exposure to the hematology-oncology unit were reviewed for clinical and epidemiological characteristics using a standardized form.

### Environmental Assessment and Sampling

Environmental assessments of the affected building and its environs were performed. Infection prevention staff and facility engineers participated in open-ended interviews about *Legionella* prevention practices. When the affected building opened in 2009, the hospital had an established *Legionella* water management program consisting of annual environmental sampling. However, the program did not contain key elements from legionellosis prevention guidelines,^10^ including routine testing of other water parameters (eg, temperature, pH, and chlorine levels) or clinician education.

Based on the environmental assessment, 64 bulk water samples (1 liter each) and biofilm swab samples were collected from 30 locations for *Legionella* culture according to established methods.^10^ Sampled sites included the point of entry for municipal water flowing into the affected building, water circulating in the 140°F supply and 120°F return loops of the central water system, and point-of-use faucets in patient care areas in the hematology-oncology unit and other units in the affected building. Water temperature, pH, and total chlorine residual were measured at sites throughout the potable water system. Records of all previous environmental sampling for *Legionella* in the affected building from January 2010 through May 2014 were also reviewed.

### Laboratory Methods

All patient isolates and environmental samples were processed at the CDC’s Pneumonia Response and Surveillance Laboratory according to previously established methods.^11,12^ Monoclonal antibodies (MAbs) were used to determine whether *Legionella* isolates were *L. pneumophila* serogroup 1 (Lp1) and whether they reacted with MAb2, a marker of enhanced virulence potential.^13,14^ Multiplex polymerase chain reaction (PCR) was used to test whether non-Lp1 isolates were *L. pneumophila* or belonged to other *Legionella* species.^15^ Finally, 7-gene sequence-based typing (SBT) was performed on all Lp1 clinical isolates and a subset of 9 Lp1 environmental isolates as previously described.^16,17^ For non-*pneumophila* isolates, *mip* gene sequencing was undertaken to determine the species. *L. pneumophila* isolates from (non-Lp1) serogroups underwent slide agglutination and direct fluorescence antibody testing to determine the serogroup.

### Ethics Review

The CDC reviewed plans for this investigation which was determined to be a non-research study because it constituted an urgent public health response.

## RESULTS

### Epidemiological Results

**FIGURE 1:**
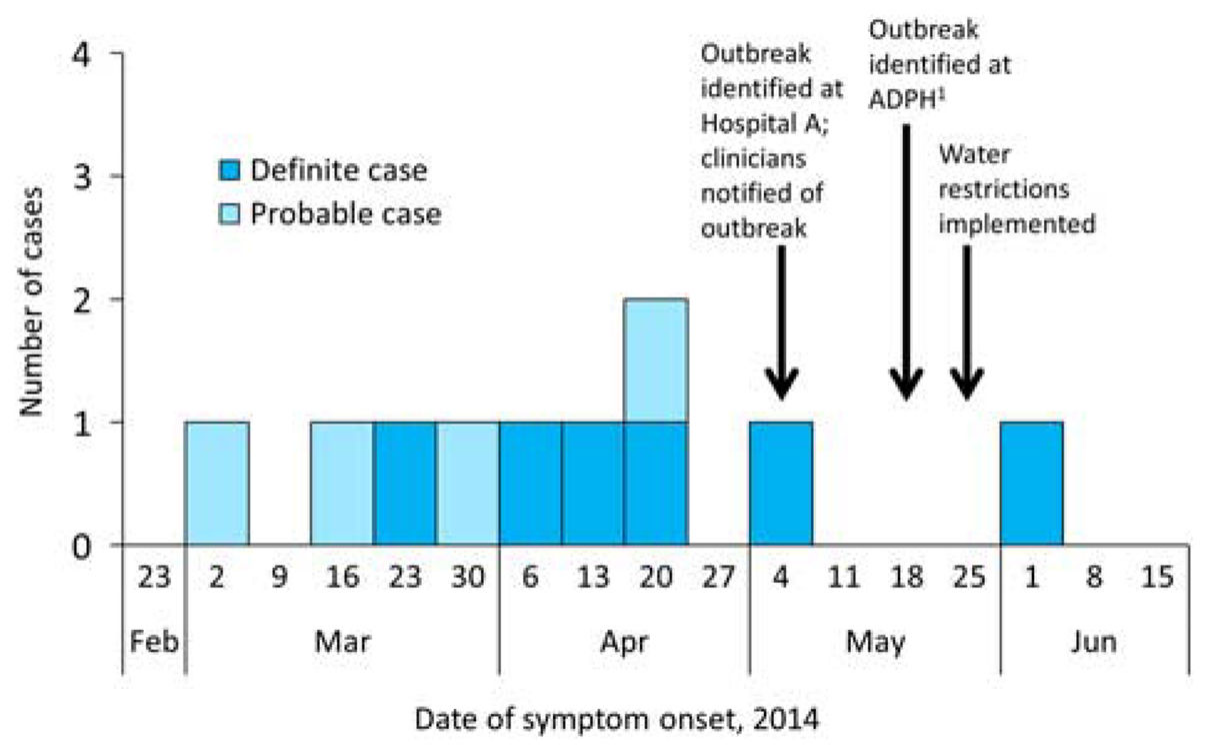
Epidemic curve of healthcare-associated cases of Legionnaires’ disease (n =10) by week of symptom onset. ADPH, Alabama Department of Public Health.^1^ All cases were reported to the respective local health departments within 1 week of positive test results.

In total, 10 cases were associated with this outbreak, with symptom onset dates between March 8 and June 3, 2014. Of these 10 cases, 9 cases were identified among the 443 inpatients who were admitted to the hematology-oncology unit for at least 12 hours between the unit opening and the beginning of the investigation. Furthermore, 1 case occurred in a visitor who stayed overnight with a relative in the same unit (Figure 1). No cases were identified among patients with exposure to other parts of the affected building or among inpatients admitted to other buildings on the hospital campus.

Case characteristics are summarized in Table 1. Of the 10 cases, 6 (60%) were classified as definitely healthcare associated, and 4 (40%) were classified as probably healthcare associated. All case patients had at least 1 medical risk factor; 1 patient had a history of a lung transplant and chronic neutropenia, the visitor had a diagnosis of chronic obstructive pulmonary disease, and the remaining 8 patients had active leukemia. A single patient was originally admitted with a new diagnosis of acute myelogenous leukemia (AML) and received the first oncologic treatment during this admission to the hematology-oncology unit.

**FIGURE 2:**
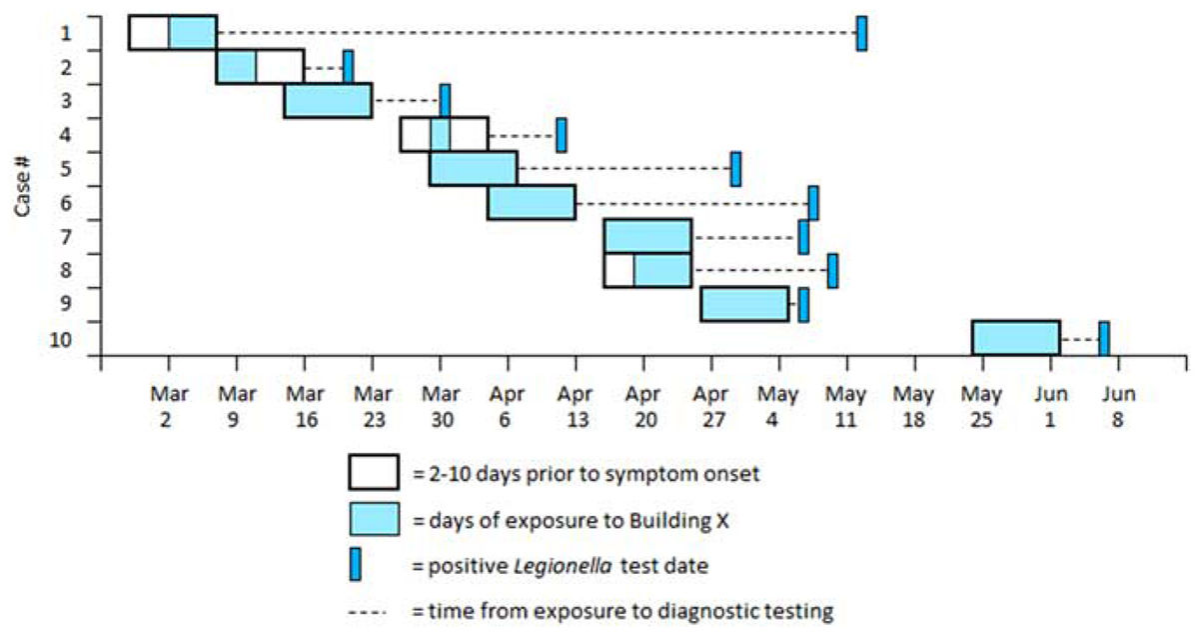
Probable incubation period (2–10 days prior to symptom onset), exposure to hematology-oncology unit, and positive *Legionella* test collection date by case patient (n = 10).

Figure 2 shows the probable window of case exposure to *Legionella* (2–10 days prior to symptom onset), the dates of exposure to the hematology-oncology unit, and the dates of *Legionella* testing. The median time between symptom onset and *Legionella* testing was 8.5 days (range, 0–65 days). The outbreak was first recognized by the hospital laboratory and infection prevention team during the first week of May, at which time all hematology-oncology unit clinicians were notified (treating clinicians were notified of test results as they became available). Subsequent testing of patients with exposure to the affected building was performed at the discretion of individual clinicians; 24 of the 89 total inpatients exposed to the unit after outbreak recognition had been tested for *Legionella* at the time of the investigation. Patients who tested positive all had healthcare-associated pneumonia consistent with LD.

### Environmental Results

The affected building received water directly from the municipal water supply. Water was distributed throughout the building via 3 independent water risers supplying floors 1–3, 4–7 (including the hematology-oncology unit), and 8–9, respectively. Each patient room was single occupancy and was equipped with 2 sinks and a shower, and each unit had several sinks for staff use as well as an ice machine connected to the building’s water system. Further assessment of the hospital campus did not identify any nearby cooling towers, and the affected building did not contain whirlpool spas, water-birth facilities, patient bathtubs, decorative fountains, or other obvious sources of aerosolized water.

At points of use, the median hot water temperature after 2 minutes was 102.5°F (range, 92.5°F–112.4°F) and median pH was 7.5 (range, 7.0–8.5). The total chlorine in the municipal water at the entrance to the affected building was measured at 1.2 ppm (a level thought to inhibit the growth of *Legionella*), but the chlorine residual in the cold water dropped at most points of use, including to undetectable levels (<0.1 ppm) in some hematology-oncology unit rooms. Total chlorine in the hot water system supplying the unit was undetectable at 7 of 12 points of use.

Records from the *Legionella* water management program revealed that no *Legionella* species were isolated from the affected building until 2012, when *Legionella* species were isolated from samples taken on floors 1, 8, and 9 (supplied by separate water risers). In 2013 and 2014, *Legionella* species including Lp1 were detected at sites supplied by the same water riser as the hematology-oncology unit, even though no testing was performed on the unit itself. Following identification of the outbreak by the hospital, additional sampling followed by superheating and flushing took place on May 7–9. The referral laboratory where these samples were cultured forwarded 5 isolates from floors 4–6 to the CDC for subtyping.

### Laboratory Results

**FIGURE 3:**
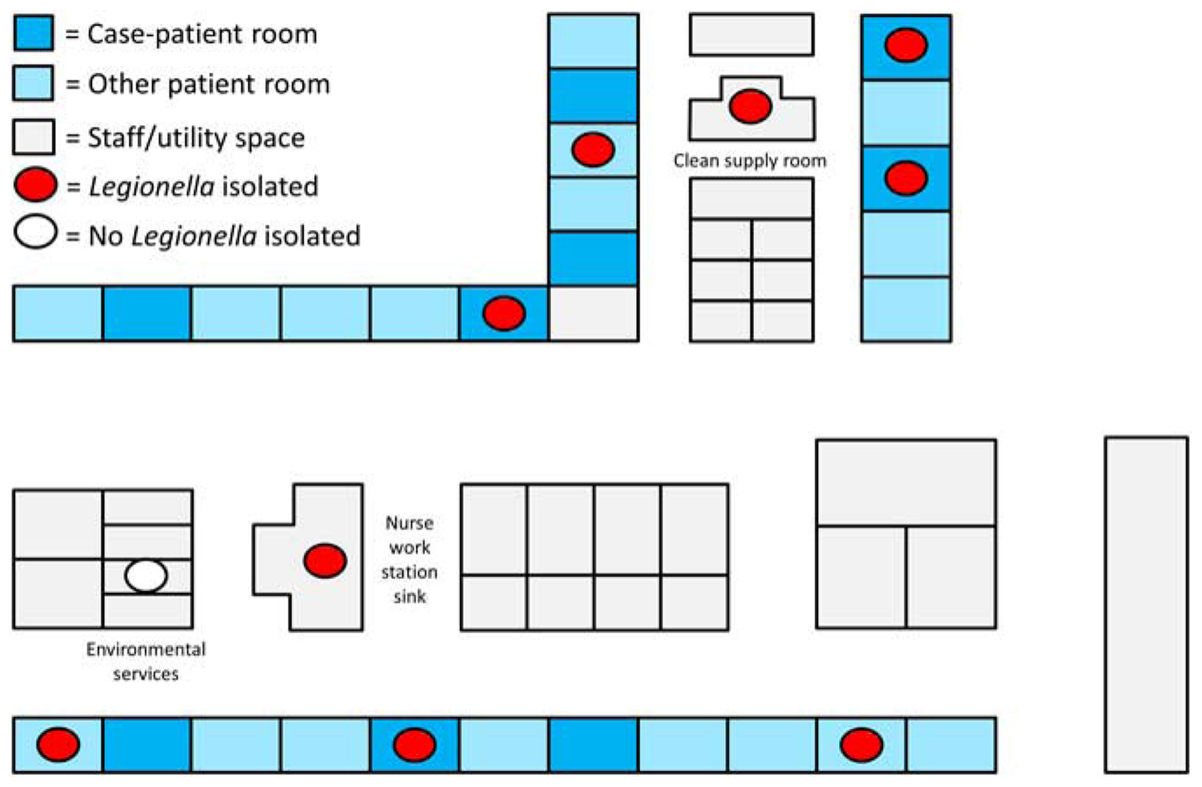
Schematic of the hematology-oncology unit showing the sites of positive environmental sampling results (figure not to scale). Of 27 rooms, 9 were occupied by case patients for at least 1 night; *Legionella* species were isolated from points-of-use in each of the 4 case patient rooms from which samples were obtained.

*Legionella* species were cultured from 21 of 30 sites (70%) sampled during the environmental investigation; legionellae were not recovered from the 2 sites that were not supplied by the second water riser (a sink in a neighboring building and water entering the affected building). Of 10 point-of-use sites on the hematology-oncology unit, 9 (90%) showed *Legionella* growth (Figure 3), including all 4 of the case patient rooms sampled. Multiple species of *Legionella* were recovered, including Lp1, Lp13, and several non-*pneumophila* strains; however, Lp1 was identified at all sites showing *Legionella* growth. MAb testing identified both MAb2-positive and MAb2-negative Lp1 strains; 15 sites (72%) showed both types. Sequence typing was performed on 9 Lp1 isolates from 7 sites (7 of these isolates [78%] were MAb2-positive), on the 3 Lp1 clinical isolates, and on 3 Lp1 isolates collected from the affected building prior to the investigation. All Lp1 isolates had identical sequence type results (ST36).

### Outbreak Response and Remediation

Water restrictions (limiting contact with the affected building’s potable water to washing visibly soiled hands) were implemented on May 25 for patients, visitors, and staff. Bottled water was provided for drinking and hygiene activities, and alcohol-based hand sanitizer was provided for routine hand cleansing; distilled water was routinely used with respiratory equipment prior to this outbreak. These restrictions were lifted once 0.2–μm point-of-use filters were obtained for all sinks, shower-heads, and ice machines in the affected building. Remediation of the potable water system was initiated once environmental samples were obtained and consisted of superheating each of the 3 water-riser systems to 160°F, flushing, and hyperchlorination (a chlorine injection system was installed for emergency remediation). Ongoing monitoring of chlorine at points of use and follow-up sampling with subsequent remediation as needed were advised. Only 1 case occurred with symptom onset after the implementation of water restrictions and remediation, but her incubation period overlapped with the period before these interventions.

## DISCUSSION

Our investigation strongly implicates the potable water system as the likely source of this outbreak; Lp1 strains isolated from water on the unit were indistinguishable from all 3 clinical specimens by SBT. Strain, environmental, and host factors likely contributed to the outbreak. ST36 is considered highly virulent based on its association with previous outbreaks, including the landmark LD outbreak in Philadelphia in 1976.^1,18^ Environmental risk factors for contamination of the potable water system included water temperatures favoring *Legionella* amplification (77–108°F^10,19–21^), inadequate biocide (eg, low chlorine^21–23^), the recent internal construction on the unit,^24–26^ and probable water stagnation in the distal piping and water fixtures in the weeks between completion of the unit’s plumbing and its opening.^21^ These findings are consistent with a recent review of environmental factors contributing to outbreaks of legionellosis.^27^ Host factors also contributed. While any hospitalized patient should be considered at increased risk for LD, hematology-oncology patients may be at comparatively greater risk based upon immune compromise resulting from active leukemia, recent chemotherapy, and systemic steroid use.^7,28,29^ Interestingly, case patients had strikingly similar clinical histories: 8 (80%) had active leukemia (including 7 with AML), all had received chemotherapy during their admission and were severely neutropenic. No patients received antibiotics recommended for the treatment of legionellosis^30^ in the 10 days prior to symptom onset, but 7 (70%) had systemic steroid exposure during the same interval. The single visitor also had risk factors for LD, underscoring the importance of considering visitors when implementing prevention measures in a healthcare-associated outbreak.

Although >25% of patients admitted to the hematology-oncology unit were ultimately tested for *Legionella*, all patients who tested positive by urine antigen or culture had symptoms of healthcare-associated pneumonia within 2–10 days of exposure to the unit (ie, no “subclinical” or “asymptomatic” cases were observed). Furthermore, 2 patients tested positive after symptom resolution, which is consistent with reports of patients shedding *Legionella* antigen in the urine for months after a severe infection.^31^ Patients with immune compromise may be more likely to experience prolonged antigen excretion.^32,33^ Our findings support the consideration of a positive urine antigen test as highly specific for clinical illness.^34^

No cases occurred with exposure exclusively after the implementation of water restrictions and installation of 0.2 μm point-of-use filters. Although it is not possible to distinguish the individual contribution of the filters to the resolution of this outbreak, the results of this investigation are consistent with previous reports that 0.2–μm point-of-use filters are effective at preventing the passage of *Legionella* species.^35,36^ Current guidelines^37^ recommending water restrictions explicitly for patients on transplant units should be broadened to include all hospitalized patients in an outbreak setting.

Prompt recognition of LD outbreaks in healthcare settings is essential to protecting vulnerable patients. Multiple factors contributed to the 2-month delay in the detection of this outbreak, illustrating several important lessons for health departments, healthcare facilities, and clinicians. First, although the hospital had a *Legionella* water management program, providers were not routinely notified of positive environmental testing results. Consequently, clinicians may have been less likely to include diagnostic testing for LD in their initial management of patients with healthcare-associated pneumonia, resulting in a delay in diagnosis and ineffective empiric antibiotic treatment. Regular clinician education should be an integral part of a hospital’s *Legionella* water management program; a toolkit to help facilities develop an effective program is now available on the CDC website.^38^ The CDC recommends *Legionella* testing for patients who develop pneumonia at least 48 hours after admission to a healthcare facility,^37^ particularly when *Legionella* species have been previously identified in the potable water. Testing by both urine antigen test and respiratory culture is recommended to maximize case detection.^39^ Second, some cases were misclassified as community acquired rather than healthcare associated at local health departments.

Review of interview documentation revealed that several case patients answered questions about symptom onset based upon their leukemia rather than LD. When interviewing patients with comorbidities, it is important to clarify the reason for the interview and to attempt to identify the onset of respiratory signs and symptoms because other symptoms of LD (nausea, malaise, fever) may not be sufficiently specific. Third, the report of a single definitely healthcare-associated LD case (eg, a patient with continuous exposure to a healthcare facility during the entire 10-day incubation period) should prompt public health action. The CDC recommends responding to a single definitely healthcare-associated LD case with a full investigation. Finally, public health surveillance should include reporting requirements to reliably identify when 2 reported cases share a common exposure to a healthcare facility. In this outbreak, all cases were reported to the appropriate local public health authorities, but their common exposure to a single hospital was initially missed. This may have occurred in part because the case patients were residents of multiple jurisdictions, consistent with the hospital’s statewide patient population. Furthermore, when a healthcare facility or laboratory identifies 2 or more cases with a common exposure, public health authorities should be notified immediately.

This investigation has several limitations. First, by limiting our case definition to laboratory-confirmed cases, we could not quantify the scope of the outbreak. We did not attempt to identify “possible” cases (patients who developed pneumonia following exposure to the affected building but who did not undergo timely *Legionella* testing), as healthcare-associated pneumonia commonly occurs among hematology-oncology inpatients due to multiple etiologies. In addition, while we hypothesize that clinical features such as leukemia, neutropenia, and steroid use contributed to increased risk for LD, we did not collect data on a “control” population to assess the magnitude of risk posed by various clinical factors. Finally, although transmission appeared to cease after the implementation of water restrictions and filter placement, it is not possible to determine the individual contribution of these measures, given that other remediation efforts were implemented simultaneously.

In conclusion, we describe an outbreak of healthcare-associated LD among a vulnerable patient population. Water restrictions and point-of-use filters may help to halt transmission, and their immediate implementation should be considered to protect susceptible patients when potable water is a suspected outbreak source. Healthcare facilities should employ a *Legionella* water management program and should follow established prevention guidelines for building water systems,^10^ with attention to key requirements for routine water quality monitoring, corrective action, and documentation. The program should include clinician education regarding the presence of *Legionella* species in the potable water system.^38^ To improve the timeliness of outbreak detection in healthcare facilities, we recommend prompt reporting of healthcare-associated legionellosis to public health authorities, followed by investigation when ≥2 cases share a common facility exposure or when a single case has continuous facility exposure throughout the entire 2–10-day incubation period.

## Figures and Tables

**TABLE 1:** Characteristics of Legionnaires’ Disease Case Patients (n = 10) With Exposure to a Hospital Hematology-Oncology Unit — Alabama, 2014.

Case Characteristics	No. (%)^a^	Median (range)
Demographics		
Age, y		58.5 (43–85)
Male	5 (50)	
White race	7 (70)	
Hispanic ethnicity^b^	0 (0)	
Signs and symptoms of Legionnaires’ disease		
Fever (temperature ≥100.4°F)	10 (100)	
Shortness of breath	9 (90)	
Cough	8 (80)	
Diarrhea	5 (50)	
Nausea	3 (30)	
Confusion/altered mental status	2 (20)	
Hypoxia (oxygen saturation <90%)	8 (80)	
Chest imaging suggestive of pneumonia^c^	10 (100)	
Positive *Legionella* urinary antigen test	10 (100)	
Positive *Legionella* respiratory culture^d^	3 (43)	
Medical history and risk factors		
Any known medical risk factor^e^	10 (100)	
Current or former smoker	5 (50)	
Alcohol abuse^f^	2 (20)	
Active leukemia diagnosis	8 (80)	
Acute myeloid leukemia	7 (88)	
Chronic lymphocytic leukemia	1 (13)	
Received chemotherapy^g^	8 (100)	
Received radiation^g^	0 (0)	
Leukopenia^g^,^h^	9 (100)	
Antibiotic exposure prior to symptom onset^i^,^j^	1 (10)	
Systemic steroid exposure prior to symptom onset^i^,^k^	7 (70)	
Exposure history		
Days of exposure to hematology-oncology unit^l^		9 (2–9)
Any invasive medical procedure^m^	3 (30)	
Outcome and complications		
Survived to hospital discharge	8 (80)	
Required ICU admission	7 (70)	
Required mechanical ventilation	6 (60)	

NOTE. ICU, intensive care unit.

a Data were available for all 10 case patients except where indicated.

b Ethnicity data were available for 3 case patients.

c Defined as a chest x-ray or computed tomography scan with evidence of a new consolidation or infiltrate.

d Respiratory cultures were obtained for 7 case patients.

e Clinical risk factors include chronic lung disease, immune suppression from a medical condition (eg, diabetes, cancer, kidney failure), and immune suppression from medications.

f Alcohol abuse was considered to be present if it was documented in the chart under “past medical history” or if social history indicated >14 alcoholic drinks/week (women) or >21 alcoholic drinks per week (men).

g At any point during the admission where exposure to *Legionella* likely occurred.

h Defined as white blood cell count (WBC) < 4,000 cells/μL; data were available for the 9 inpatients only, and of these, 8 patients had a white cell differential available and all were neutropenic, with median absolute neutrophil count of 22 cells/μL (range, 0–43).

i During the 10 days prior to symptom onset.

j Excludes antibiotics with no known *in vivo* activity against *Legionella* species (eg, β-lactams, aminoglycosides, vancomycin); 1 case patient was prescribed an antibiotic with potential activity against *Legionella* (trimethoprim [160 mg]-sulfa-methoxazole [800 mg], 1 tablet 3 days a week), and she received 2 doses over 3 days in the 10 days prior to her symptom onset.

k Systemic steroids included prednisone, methylprednisolone, hydrocortisone, and dexamethasone.

l During the 2–10 days prior to symptom onset.

m Procedures included bone marrow biopsy (n = 2), Hickmann catheter placement (n = 1), and lumbar puncture (n = 1).
